# HLH-30/TFEB modulates autophagy to improve proteostasis in Aβ transgenic *Caenorhabditis elegans*

**DOI:** 10.3389/fphar.2024.1433030

**Published:** 2024-08-30

**Authors:** Hongru Lin, Chen Zhang, Yehui Gao, Yi Zhou, Botian Ma, Jinyun Jiang, Xue Long, Nuerziya Yimamu, Kaixin Zhong, Yingzi Li, Xianghuan Cui, Hongbing Wang

**Affiliations:** ^1^ Institute for Regenerative Medicine, Shanghai East Hospital, School of Life Sciences and Technology, Tongji University, Shanghai, China; ^2^ Department of Anesthesiology, The First Affiliated Hospital of Naval Medical University, Shanghai, China; ^3^ Tongji Alpha Natural Medicine Research Institute, Tongji University, Shanghai, China

**Keywords:** HLH-30, amyloid beta, autophagy, HLH-30/TFEB activators, *Caenorhabditis elegans*

## Abstract

Alzheimer’s disease (AD) is a complex neurodegenerative disease that affects elderly individuals, characterized by senile plaques formed by extracellular amyloid beta (Aβ). Autophagy dysfunction is a manifestation of protein homeostasis imbalance in patients with AD, but its relationship with Aβ remains unclear. Here, we showed that in Aβ transgenic *Caenorhabditis elegans,* Aβ activated the TOR pathway and reduced the nuclear entry of HLH-30, leading to autophagy dysfunction characterized by autophagosome accumulation. Then, utilizing RNA-seq, we investigated the regulatory mechanisms by which HLH-30 modulates autophagy in *C. elegans.* We found that HLH-30 elevated the transcript levels of v-ATPase and cathepsin, thus enhancing lysosomal activity. This led to an increase in autophagic flux, facilitating more pronounced degradation of Aβ. Moreover, HLH-30 reduced the level of ROS induction by Aβ and enhanced the antioxidant stress capacity of the worms through the *gsto-1* gene. Additionally, we identified two HLH-30/TFEB activators, saikosaponin B2 and hypericin, that improved autophagic flux, thereby enhancing protein homeostasis in *C. elegans*. Overall, our findings suggested that HLH-30/TFEB plays a key role in modulating autophagy and can be considered a promising drug target for AD treatments.

## Introduction

Alzheimer’s disease (AD) is characterized by the accumulation of amyloid beta (Aβ) peptides and hyperphosphorylated tau (p-Tau) protein in senile plaques and neurofibrillary tangles, respectively, which cause neurotoxicity and neuron loss, leading to memory and learning impairments (Silva et al., 2019). Therefore, the removal of misfolded proteins plays a significant role in maintaining protein homeostasis in the treatment of AD (Lukiw, 2022). The autophagy–lysosome pathway (ALP) is a cellular pathway in which cells utilize lysosomes to degrade long-lived proteins and organelles. The inability of the ALP to fully clear misfolded proteins has been recognized as important in the pathophysiology of AD (Cheng et al., 2023). Correspondingly, autophagy dysfunction is prevalent in AD patients (Ghavami et al., 2014); however, the mechanisms underlying this dysfunction are poorly understood.

The transcription factor EB (TFEB, known as HLH-30 in *Caenorhabditis elegans*), a member of the MiT/TFE family of transcription factors, is a key regulator of ALP (Martini-Stoica et al., 2016). It is translocated into the nucleus to coordinate the expression of autophagy and lysosomal target genes (Napolitano and Ballabio, 2016). The subcellular localization and activity of TFEB are regulated by mammalian target of rapamycin (mTOR)-mediated phosphorylation, which occurs at the lysosome surface (Zhu et al., 2019a). Phosphorylated TFEB is retained in the cytoplasm, whereas dephosphorylated TFEB translocates to the nucleus to induce the transcription of target genes (Puertollano et al., 2018). In AD patients, the level of TFEB in the nucleus is significantly reduced in cells in the hippocampus (Wang et al., 2016), which is the main brain region where autophagy dysfunction occurs (Nixon and Yang, 2011). Therefore, inhibition of TFEB nuclear translocation may be closely related to autophagy dysfunction. In our previous work, we reported impaired autophagic flux and abnormally elevated LET-363 (a mammalian ortholog of TOR) in Aβ transgenic *Caenorhabditis elegans* (*C. elegans*) (Lin et al., 2022). It has been reported that LET-363 can affect the nuclear localization of HLH-30 (Nakamura et al., 2016). However, the correlation between the aberrant upregulation of LET-363 expression and impaired autophagic function in Aβ transgenic worms has not yet been tested.

Accumulating studies have suggested that targeting TFEB is promising for the treatment of AD (Gu et al., 2022). However, there are very few effective and specific small molecule TFEB activators at present. The main known activators of TFEB are mostly mTOR inhibitors (Song et al., 2016). mTOR is well-known as a major regulator of cell growth and metabolism and is involved in a wide range of biological functions. Therefore, identifying TFEB-specific activators would be an important step in developing better therapeutics.

In this study, we determined that Aβ could activate TOR activity, inhibit the nuclear localization of HLH-30, and block the fusion of autophagosomes with lysosomes, thus leading to autophagy dysfunction in *C. elegans*. In addition, RNA sequencing to investigate the molecular mechanisms by which HLH-30 regulates autophagy revealed a close association between lysosomal function and autophagy dysfunction. Finally, we searched for HLH-30/TFEB-specific activators by molecular docking and found two active compounds, saikosaponin B2 and hypericin, that could specifically promote the nuclear entry of HLH-30 without inhibiting TOR activity, thereby enhancing autophagy and facilitating the degradation of Aβ.

## Materials and methods

### Strains and maintenance conditions

All strains used in this study are detailed in Supplementary Table S1. All strains were maintained and cultured under standard conditions at 20°C using *E. coli* OP50 as a food source, except CL4176, which was maintained at 16°C according to standard procedures.

### Transgenic strain construction

The plasmid pMH878 (*lgg-1*p::mcherry::gfp::*lgg-1*) was donated by Malene Hansen (Chang et al., 2017). PHX3392, PHX3636 and PHX6883 were generated by SunyBiotech at our request. We knocked out the first four exons of the *hlh-30* gene to construct PHX6883. Other strains were obtained by hybridization.

### Gene expression analysis by quantitative PCR (qPCR)

Total RNA was extracted using TRIzol A+ and reverse transcribed into cDNA. The expressed genes were amplified in triplicate and quantified by PCR using SYBR Green PCR Mix (B21702, Bimake, China) with a Roche LightCycler system. The data were analyzed using the 2^−ΔΔCT^ method (Lin et al., 2022). The primer list has been added to Supplementary Table S2.

### Paralysis assay

Aβ transgenic CL4176 worms were maintained on NGM at 16°C and synchronized. L1 animals were treated with drugs or RNAi-engineered bacteria (or left untreated) for 36 h and then transferred to 23°C for transgene induction. This temperature shift stimulated Aβ expression, causing Aβ aggregation and leading to paralysis. Scoring began 27 h after the temperature shift, and the worms were considered paralyzed if they failed to move their bodies when touched and produced a “halo” of cleared bacterial lawn because they moved only their heads while feeding. We used PT_50_ (the time interval from the onset of paralysis at which 50% of the worms were paralyzed) as an index (Wu et al., 2006). For example, the PT_50_ of 4.2 h for the control was obtained by subtracting the time at which paralysis was first observed (29 h) from the time when 50% of the worms were paralyzed (33.2 h). The assay was performed at least three times. Each individual group contained more than 30 worms. Statistical analysis was conducted with GraphPad Prism 6.0 software, and *p* values were calculated using the log-rank test.

### LysoTracker red staining

The acidophilic dye LysoTracker Red (C1046; Beyotime Biotechnology, Shanghai, China) was used at a final concentration of 15 μM. Synchronized strains maintained at 20°C were treated with LysoTracker from the L1 to the young adult stage and then transferred to 25°C for transgene induction. After 24 h, the worms were washed twice with fresh M9 (without LysoTracker) and imaged using a Revolve microscope (Imanikia et al., 2019).

### RNA interference (RNAi)

RNAi experiments were performed according to a previously reported protocol. In brief, RNA was delivered to the worms by feeding, gravid adults were bleached, and the eggs were placed on NGM dishes supplemented with 1 mM isopropyl β-D-1-thiogalactopyranoside (IPTG). An HT-115(DE3) bacterial colony containing L4440 or the target gene plasmid was inoculated in LB broth supplemented with 100 μg/mL ampicillin and 12.5 μg/mL tetracycline and grown for 8 h in a 37°C shaker. The bacteria were plated on an NGM dish containing IPTG 1 h prior to the addition of the worms. The synchronized strain was grown on RNAi NGM plates from the egg stage to the adult stage for two generations. Synchronized L1 worms were treated with or without drugs for paralysis assays as described above (Zhang et al., 2016).

### Measurement of reactive oxygen species (ROS)

Endogenous ROS levels were measured using 2′,7′-dichlorofluorescein diacetate (H2DCF–DA), which reacts with endogenous ROS to generate a fluorescent product (Yang et al., 2018). Worms were treated as in the paralysis assay, and ROS were measured at the point of paralysis. The cells were then incubated with 50 μM H2DCF–DA for 30 min at 37°C, and the fluorescence intensity was measured at excitation and emission wavelengths of 485 and 535 nm, respectively. The assay was performed in triplicate.

### Oxidative stress resistance assay

Synchronized worms were cultured to the young adult stage. Then, the worms were transferred to a new 96-well plate containing 1 mM H_2_O_2._ The worms were monitored every 12 h and scored as dead when they no longer moved through the liquid medium (Chen et al., 2018).

### HLH-30 translocation assay

The transgenic strain was used to observe the intracellular localization of HLH-30. Age-synchronized L1 worms were treated with or without 100 μM rapamycin for 3 days, mounted on 2% agarose pads and visualized under a fluorescence microscope (Revolve FL, Echo Laboratories, United States) for GFP localization. The nuclear accumulation of HLH-30::GFP was quantified by ImageJ (Visvikis et al., 2014). We use 4% paraformaldehyde as anesthetic. The assay was performed three times independently.

### Western blotting

The worms were synchronized, and eggs were allowed to hatch and develop to the L4 stage on NGM plates at 20°C. Then, the temperature was increased to 25°C for 24 h. The worms were then collected from the plates with M9 buffer and washed twice to remove bacteria. Nuclear protein extraction was performed using a nuclear extract kit (Active Motif). The samples were heated at 100°C in sample loading buffer for 10 min and centrifuged at 10,000 × g for 10 min (Sangha et al., 2015). The collected supernatant was boiled with loading buffer at 100°C for 5 min before being loaded into the gel, which also included a 10–180 kDa protein molecular weight marker (PR1910, Solarbio, China) (Zhu et al., 2019b). The samples were run at 40 V for 40 min on a stacking gel and at 80 V for 120 min on a separating gel. The gel was then transferred to a polyvinylidene fluoride membrane using 20% methanol transfer buffer at 100 V for 1 h. The membranes were then blocked in Tris-buffered saline with Tween 20 + 5% skim milk for 1 h. The level of phosphorylated RSKS-1 protein was detected with an anti-phospho-p70 S6 kinase antibody (dilution 1:2,000; 9205, Cell Signaling Technology), with an anti-β-actin antibody (dilution 1:2,000; 60008, Proteintech) as a control. The RSKS-1 protein level was detected with an anti-p70 S6 kinase antibody (1:2,000 dilution; 9202, Cell Signaling Technology). The Aβ protein level was detected with the 6E10 monoclonal antibody (dilution 1:500; 803014, BioLegend). The protein level of mCherry-GFP-LGG-1 was detected with an anti-GFP antibody (dilution 1:500 Beyotime Technology). Histone H3 (1:2,000 dilution; ab1791 dilution, Abcam) served as an internal reference protein for the nuclear protein experiments. The membrane was incubated with the primary antibody overnight at 4°C and then with the secondary antibody for 2 h at room temperature. Images were captured using a Chemiscope 3400 mini Western blot imaging system (Amersham Imager 600, GE, United States). The mean densities of the Aβ bands were analyzed using ImageJ software.

### Hybridization experiment

Five to eight worms in the L3 stage were selected and heat shock cultured for 3.5 h at 33°C, after which they were transferred to 20°C for further culture. A number of males could be found among the offspring. The male worms were hybridized with hermaphroditic worms, and homozygous worms were identified by agarose electrophoresis screening (Fay, 2006).

### Molecular docking

Core targets (large molecular receptors) and the corresponding active compounds (small molecule ligands) were selected for molecular docking analysis. The 3D structures of the core targets were downloaded from the PDB database (https://www.rcsb.org/). The 2D structures of the corresponding active compounds were downloaded from the PubChem database (https://pubchem.ncbi.nlm.nih.gov/). The raw files of the core targets and the corresponding active components were processed with the AutoDock (version 4.2) tool (Morris et al., 2009) and converted to PDBQT format for molecular docking. Binding activity was expressed as binding energy, with lower bonding energy corresponding to more stable docking (Morris et al., 2008). In general, a docking energy < −5 kcal/mol was considered to indicate good docking.

### RNA sequencing (RNA-seq)

The worms were synchronized (Fabian and Johnson, 1994), and their eggs were allowed to hatch and develop to the young adult stage on NGM plates at 20°C. Then, the temperature was increased to 25°C to induce Aβ expression for 24 h. Gene expression was assessed by Novo Gene Corporation (Beijing, China). Sequencing libraries were generated using the NEBNext^®^ UltraTM RNA Library Prep Kit for Illumina^®^ (NEB, United States). To preferentially select cDNA fragments 250∼300 bp in length, the library fragments were purified with the AMPure XP system (Beckman Coulter, Beverly, United States). Then, 3 µL of USER Enzyme (NEB, United States) was incubated with size-selected adaptor-ligated cDNA at 37°C for 15 min and then 5 min at 95°C. Then, PCR was performed with Phusion High-Fidelity DNA polymerase, universal PCR primers and Index (X) Primer. Finally, the PCR products were purified (AMPure XP system), and library quality was assessed on an Agilent Bioanalyzer 2100 system. Clustering of the index-coded samples was performed on a cBot Cluster Generation System using the TruSeq PE Cluster Kit v3-cBot-HS (Illumina) according to the manufacturer’s instructions. After cluster generation, the libraries were sequenced on an Illumina NovaSeq platform to generate 150 bp paired-end reads. Up- or downregulated genes were identified by filtering RNA-seq data with the following criteria: twofold change in expression level and a false discovery rate analog of *p* < 0.05. KEGG pathway enrichment analysis was performed using “clusterProfiler” with the criterion of *p* < 0.05.

### Statistical analysis

All quantified data are presented as the mean ± SD. The data were analyzed by Student’s *t* test to determine statistical significance with GraphPad Prism 6.0 software (GraphPad, La Jolla, United States). For multiple comparisons, the results were analyzed by one-way analysis of variance (ANOVA). *p* < 0.05 was considered to indicate statistical significance.

## Results

### Aβ expression affects TOR-mediated HLH-30 nuclear localization

**FIGURE 1:**
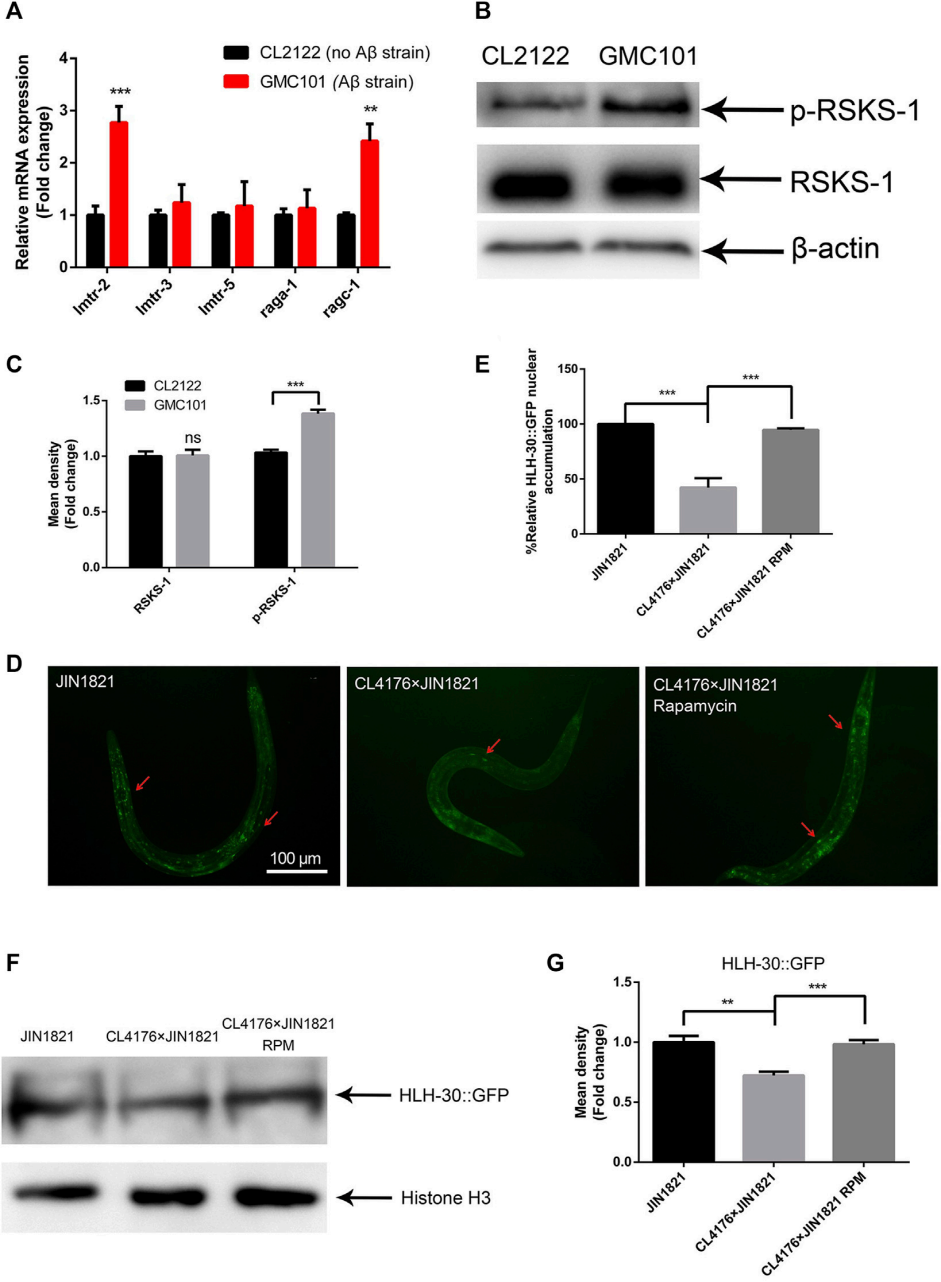
Aβ affects the nuclear localization of HLH-30 by activating mTOR. **(A)** mRNA expression of genes related to mTOR (strains GM101 and CL2122). **(B)** Representative Western blot showing phospho-RSKS-1 and total RSKS-1 in the CL2122 and GMC101 strains, three biological replicates. **(C)** Quantified Western blot gel intensities, as determined by ImageJ software. **(D)** GFP fluorescence images of HLH-30 entering the nucleus (n = 10 for each group, repeated three times). The dose of rapamycin was 100 μM. **(E)** Fluorescence signal ratio quantification. RPM, rapamycin. The HLH-30::GFP accumulation in JIN1821 was defined as 100% for quantitative comparisons. **(F)** Representative Western blot showing the content of HLH-30::GFP in the nucleus. Each experiment was repeated three times. **(G)** Quantified Western blot gel intensities, as determined by ImageJ software. **p < 0.01; ***p < 0.001. Detailed genotypic descriptions of strains CL2122, GMC101, and JIN1821 are listed in Supplementary Material.

In previous studies, we found that both the transcription and protein levels of *let-363* were significantly increased in Aβ transgenic *C. elegans* (Lin et al., 2022). Thus, we hypothesized that Aβ expression might affect TOR activity in *C. elegans*. To test this hypothesis, we evaluated the transcript levels of *lmtr-2* and *ragc-1*, which regulate TOR activation (Charar et al., 2021; Blackwell et al., 2019), and found that they were obviously upregulated (Figure 1A), suggesting that Aβ expression induced TOR activation. To further explore the regulatory role of Aβ expression in TOR activation, we examined TOR activity by measuring the phosphorylation level of its substrate RSKS-1 (an ortholog of S6K). The results showed that RSKS-1 phosphorylation in the Aβ strain GMC101 was significantly greater than that in the control strain CL2122, while the total protein level of RSKS-1 remained unchanged (Figures 1B, C). These results suggested that Aβ expression could activate the TOR pathway. TOR activation prevents the nuclear translocation of HLH-30 (Blackwell et al., 2019). The strain JIN1821 is often used to observe the nuclear localization of HLH-30 (Visvikis et al., 2014). We crossed JIN1821 with CL4176 (a transgenic strain expressing Aβ) to observe the nuclear localization of HLH-30 in the presence of Aβ. Compared with that in the control group, the nuclear entry of HLH-30::GFP was obviously reduced in the Aβ expressing group (Figures 1D, E). Moreover, the nuclear localization of HLH-30::GFP was significantly restored after the worms were treated with the mTOR inhibitor rapamycin at 100 μM (Figures 1D, E). We also observed a significant decrease in the nuclear level of the HLH-30 protein in the Aβ group compared to that in the control group (Figures 1F, G). The above results suggested that Aβ affected the entry of HLH-30 into the nucleus by activating TOR and that this may be one of important factor causing the abnormal autophagy in Aβ transgenic worms.

### The stability of autophagic flux in Aβ transgenic worms depends on HLH-30

**FIGURE 2:**
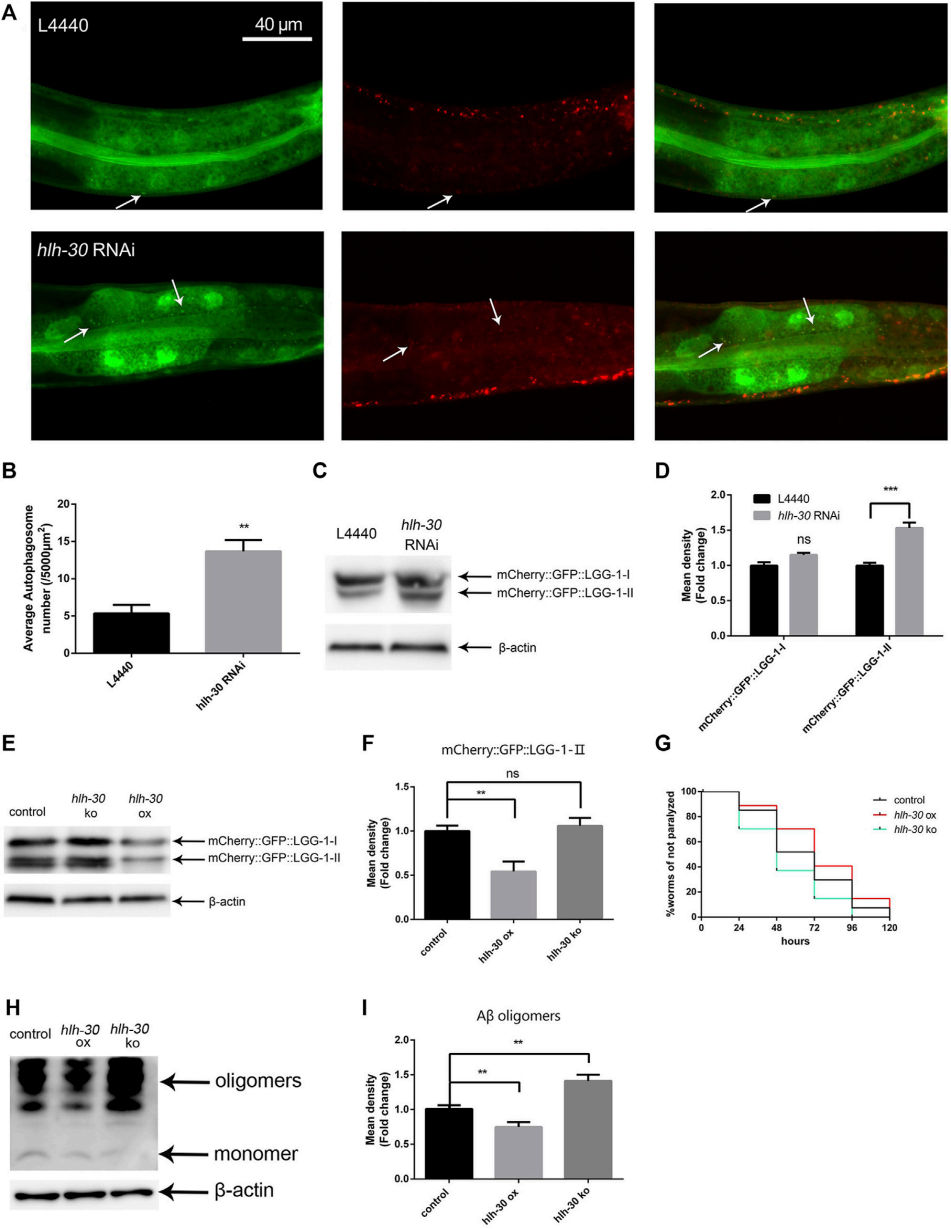
The stability of autophagic flux in Aβ transgenic worms depends on HLH-30. **(A)** Puncta formation in the PHX3636 strain as shown by fluorescence microscopy (strain PHX3636). **(B)** Scoring of punctae (n = 10 for each group, repeated three times). **(C)** Immunoblot analysis to evaluate the effects of *hlh-30* RNAi on mCherry::GFP::LGG-1 expression (strain PHX3636). **(D)** Quantified Western blot gel intensities, as determined by ImageJ software. **(E)** Effects of *hlh-30* overexpression or knockout on mCherry::GFP::LGG-1, as measured by immunoblot analysis (strains HBW003 and HBW004). “ox”, overexpression; “ko”, knockout. Each experiment was repeated three times. **(F)** Quantified Western blot gel intensities, as determined by ImageJ software. **(G)** Paralysis assay of *hlh-30*-overexpressing or *hlh-30* knockout worms (n > 30 for each group, repeated three times). **(H)** Immunoblot analysis to evaluate the effects of *hlh-30* overexpression or knockout on Aβ species. Each experiment was repeated three times. **(I)** Quantified Western blot gel intensities, as determined by ImageJ software. “ns”, not significant; **p < 0.01; ***p < 0.001. Detailed genotypic descriptions of strains PHX3636, HBW003, and HBW004 are listed in Supplementary Material.

Previous studies have shown that Aβ causes the accumulation of autophagosomes in *C*. *elegans* (Florez-McClure et al., 2007); however, the cause of this phenomenon is unknown. The transcription factor HLH-30 plays a crucial role in the autophagy‒lysosome pathway (Napolitano and Ballabio, 2016). To explore the relationship between HLH-30 and autophagy dysfunction in Aβ transgenic worms, we performed *hlh-30* RNAi on the PHX3392 (Aβ) and PHX3636 (no Aβ) strains, which express mCherry::GFP::LGG-1 (genotypes are shown in Supplementary Table S1). Thus, the autophagosomes in these strains are labeled by both mCherry and GFP (Chang et al., 2017). The number of autophagosomes in the PHX3636 (no Aβ) group was significantly greater than that in the control group after *hlh-30* RNAi treatment (Figures 2A, B). In addition, we found that the protein level of mCherry::GFP::LGG-1-Ⅱ was significantly increased (Figures 2C, D). This suggests that *hlh-30* RNAi may result in autophagy dysfunction in worms. To further investigate the mechanism by which HLH-30 regulates autophagy, we generated *hlh-30*-overexpressing (*hlh-30* ox) strains and *hlh-30 (syb6883)* knockout mutants on the basis of the Aβ strains. The use of GFP labeling in the construction of transgenic nematodes is necessary for the microscopic observation of mCherry::GFP::LGG-1. Therefore, we detected the expression level of mCherry::GFP::LGG-1 to evaluate autophagosome accumulation in worms. The results showed that overexpression of *hlh-30* alleviated the accumulation of autophagosomes induced by Aβ, while *hlh-30* knockout did not increase the accumulation of autophagosomes in Aβ worms (Figures 2E, F). Then, we compared the paralysis rates of *hlh-30*-overexpressing and *hlh-30* knockout worms. Overexpression of *hlh-30* obviously decreased the paralysis rate, while *hlh-30* knockout accelerated the paralysis rate (Figure 2G). Finally, we found that the protein level of Aβ was significantly decreased in *hlh-30*-overexpressing worms (Figures 2H, I). These results indicated that the overexpression of *hlh-30* could lead to the degradation of Aβ by enhancing autophagy.

### RNA interference of hlh-30 reduces the transcript level of syx-17 and leads to autophagosome accumulation

**FIGURE 3:**
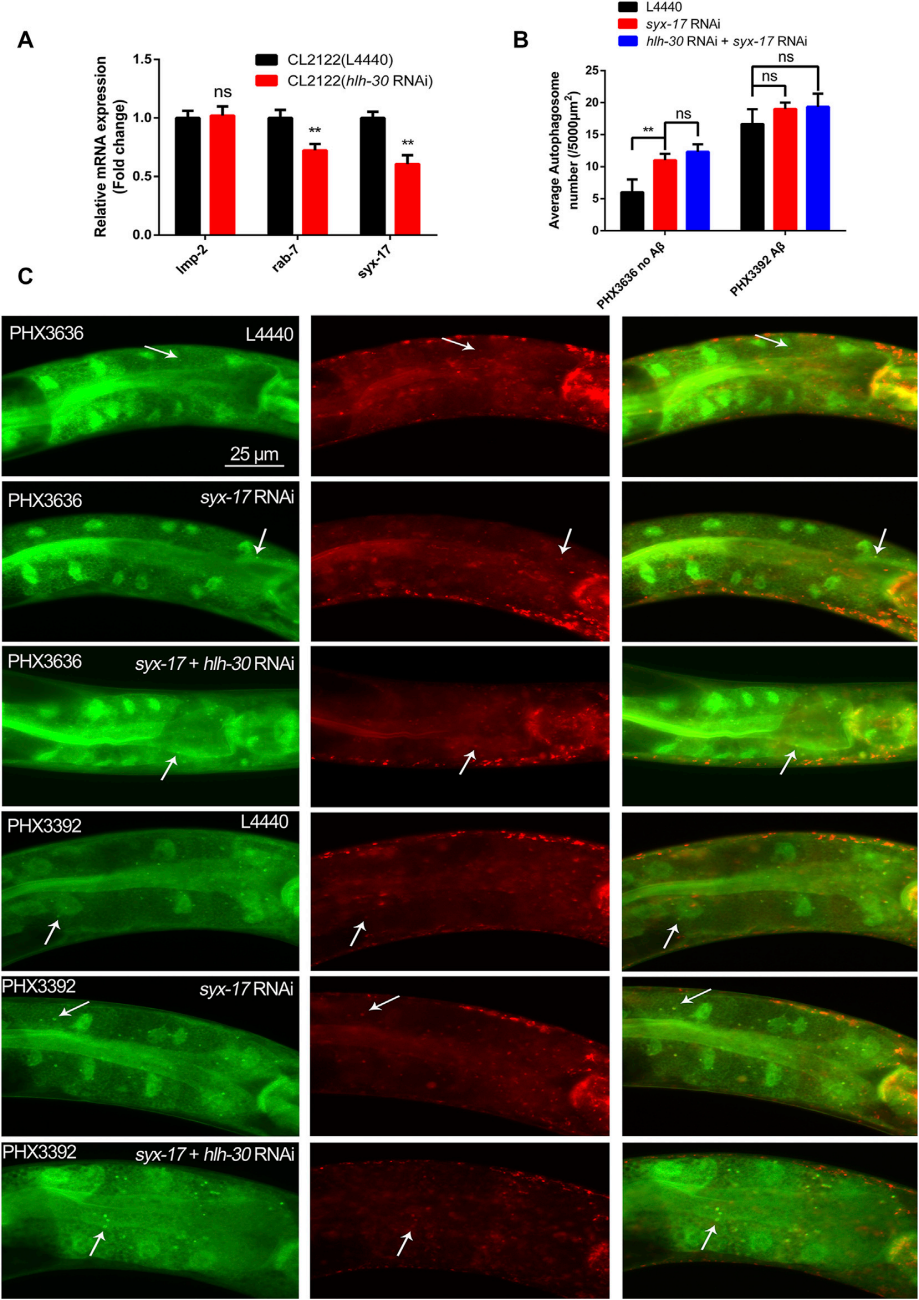
Downregulation of *syx-17* at the transcriptional level leads to the accumulation of autophagosomes in worms. **(A)** Effects of *hlh-30* RNAi on the mRNA expression of genes related to autophagosome‒lysosome fusion. **(B)** Effects of RNAi of *syx-17* or *hlh-30* on puncta formation in the PHX3636 (no Aβ) or PHX3392 (Aβ) strains, as shown by fluorescence microscopy. **(C)** Scoring of punctae (n = 10 for each group, repeated three times). “ns”, not significant; **p < 0.01. Detailed genotypic descriptions of strains PHX3636 and PHX3392 are listed in Supplementary Material.

In mammals, TFEB can regulate hundreds of ALP genes at the transcriptional level, so we hypothesized that HLH-30 may also regulate the transcription levels of ALP genes in worms. The expression levels of *rab-7* and *syx-17*, which are related to autophagosome–lysosome fusion (Zhao et al., 2021), were found to be significantly decreased by *hlh-30* RNAi (Figure 3A). It was reported that *syx-17* RNAi could block autophagosome–lysosome fusion (Gkikas et al., 2023). Therefore, we investigated the association between the reduced expression of *syx-17* and autophagy dysfunction caused by the decreased expression of HLH-30. We found that the number of autophagosomes in PHX3636 cells (without Aβ) increased significantly in response to *syx-17* RNAi (Figures 3B, C). However, autophagosome accumulation after *syx-17* and *hlh-30* dual gene RNAi was not significantly greater than that after *syx-17* RNAi in PHX3636. Similarly, the accumulation of autophagosomes in PHX3392 (Aβ) cells was not further elevated by *syx-17* RNAi (Figures 3B, C). These results indicated that the decreased expression of HLH-30 reduced the expression of *syx-17*, resulting in the abnormal fusion of autophagosomes and lysosomes in Aβ nematodes and subsequent autophagy dysfunction.

### HLH-30 maintains the stability of autophagic flux by enhancing lysosomal activity

**FIGURE 4:**
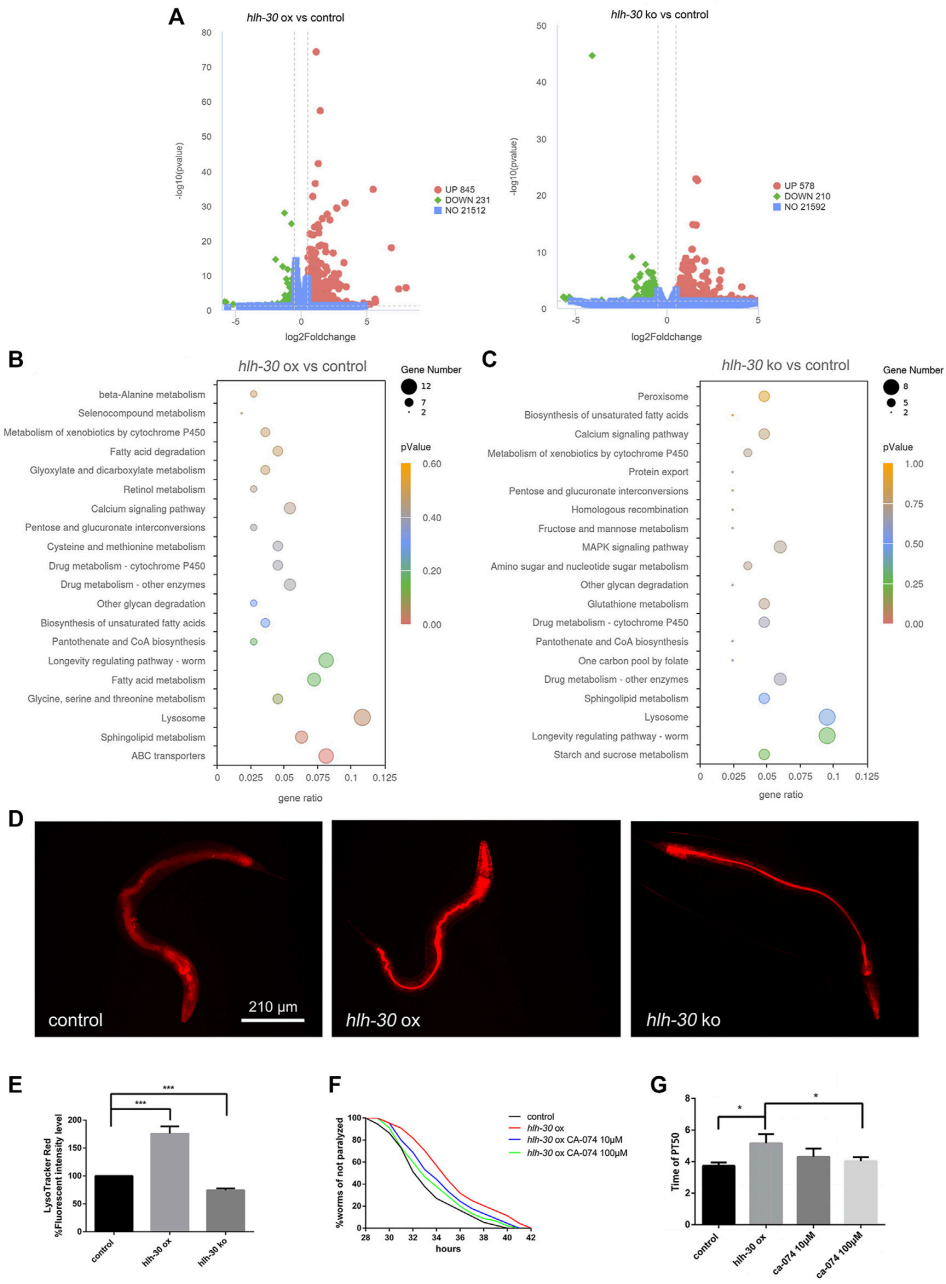
HLH-30 maintains the stability of autophagic flux by enhancing lysosomal activity. **(A)** RNA-seq differential gene volcano map. “ox”, overexpression; “ko”, knockout. **(B)** KEGG pathway enrichment of genes differentially expressed in the *hlh-30*-overexpressing strain. The *x*- and *y*-axes represent the GeneRatio and enriched KEGG pathways, respectively. The color represents the significance of the enrichment, and the bubble size represents the gene count. **(C)** KEGG pathway enrichment of genes differentially expressed in the *hlh-30* knockout strain. **(D)** Representative fluorescence images of strains stained with LysoTracker Red after Aβ induction for 24 h (n = 10 for each group, repeated three times). The worms used in this study were overexpressed and knockout strains based on GMC101. **(E)** Quantified fluorescence intensities (n = 10 for each group, repeated three times). **(F)** Overexpression of *hlh-30* delayed CL4176 paralysis in a cathepsin B-dependent manner (n > 30 for each group, repeated three times). The worms used in this study were overexpressed strains based on CL4176. **(G)** Statistical analysis of the half paralysis time of worms. *p < 0.05; ***p < 0.001.

To further explore how *hlh-30* regulates autophagy to stabilize protein homeostasis in worms, we conducted RNA-seq analysis on strains in which *hlh-30* was overexpressed or knocked out (Figure 4A). Through KEGG enrichment, we found that both the overexpression and knockout of *hlh-30* affected the transcript levels of lysosome-related genes (Figures 4B, C), which was verified by qPCR (Supplementary Figure S1). The results showed that the v-ATPase and cathepsin B genes were significantly upregulated in the *hlh-30* overexpression group (Supplementary Figure S1). To further evaluate the effect of *hlh-30* overexpression on lysosomal function, we utilized LysoTracker Red to analyze lysosome acidification. The results showed that *hlh-30* overexpression obviously enhanced lysosomal activity (Figures 4D, E).

Given the observed upregulation of cathepsin B transcription and enhancement of lysosomal activity, we hypothesized that HLH-30 promotes Aβ degradation by enhancing lysosomal activity and hydrolase expression, thereby improving protein homeostasis in worms. To further confirm the above results, we evaluated the impact of CA-074, a cathepsin-specific inhibitor (Bai et al., 2021), on the paralysis rate in *hlh-30* Aβ-overexpressing worms. The results showed that the ability of *hlh-30* overexpression to prolong paralysis was offset by the administration of CA-074 (Figures 4F, G). The above results suggested that lysosomes may be involved in the maintenance of protein homeostasis through the overexpression of *hlh-30*.

### HLH-30 maintains protein homeostasis by clearing ROS through *gsto-1*

**FIGURE 5:**
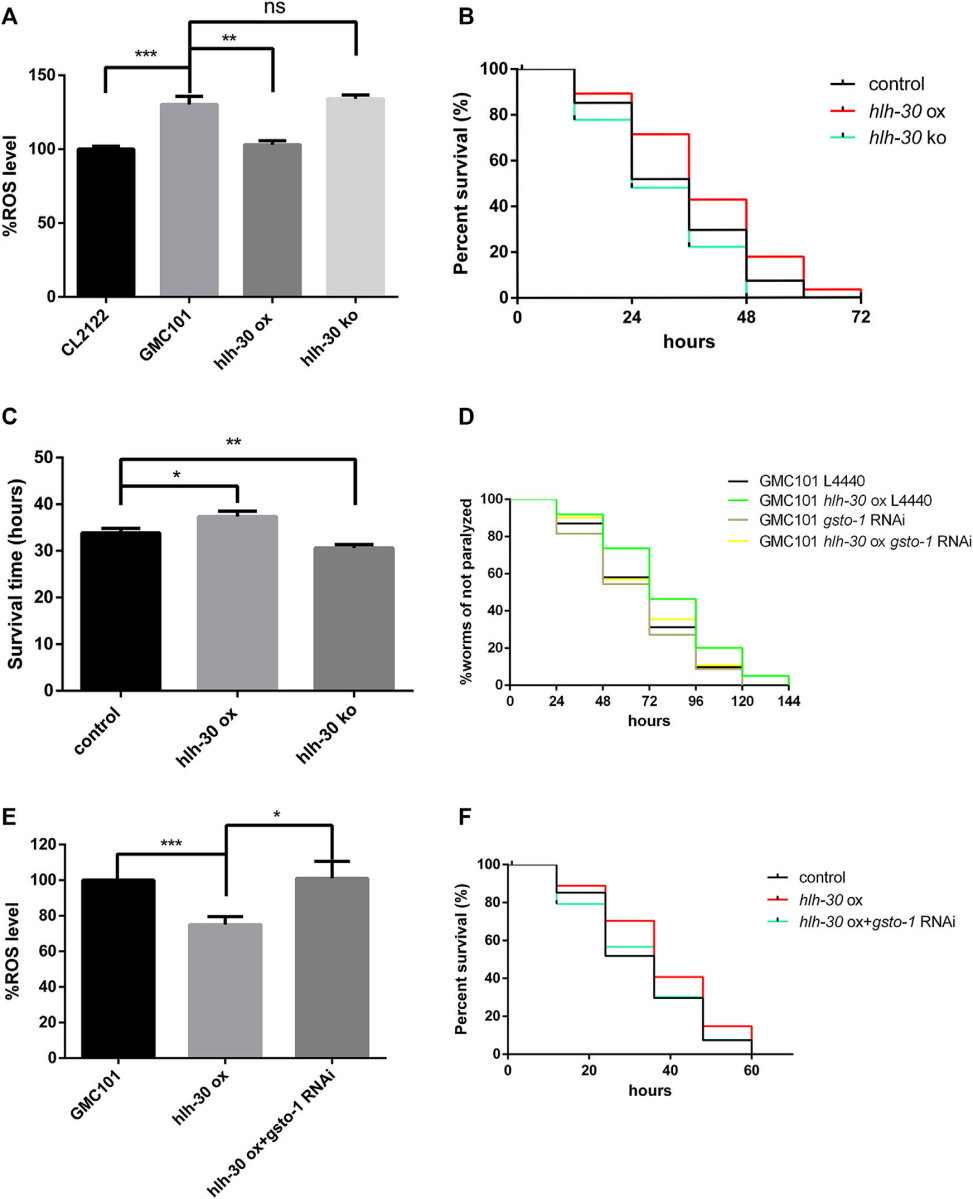
HLH-30 maintains protein homeostasis by clearing ROS through *gsto-1*. **(A)** Levels of ROS in transgenic worms (n > 30 for each group, repeated three times). “ox”, overexpression; “ko”, knockout. The worms used in this study were overexpressed strains based on GMC101. **(B)** Evaluation of the antioxidant capacity of transgenic worms (n > 30 for each group, repeated three times). The worms used in this study were overexpressed strains based on CL4176. **(C)** Survival time histogram under oxidative stress induced by hydrogen peroxide. **(D)** Paralysis assay of RNAi worms (n > 30 for each group, repeated three times). **(E)** Levels of ROS in transgenic worms (n > 30 for each group, repeated three times). **(F)** Evaluation of the antioxidant capacity of the transgenic worms (n > 30 for each group, repeated three times). “ns”, not significant; *p < 0.05; **p < 0.01; ***p < 0.001.

In previous studies, we found that Aβ can cause severe oxidative stress, manifested by a large increase in reactive oxygen species (ROS) (Lin et al., 2022). Through an ROS assay, we found that ROS levels were significantly decreased by *hlh-30* overexpression and increased in *hlh-30(syb6883)* mutants (Figure 5A). Furthermore, the survival time of *hlh-30*-overexpressing worms in an oxidative stress environment induced by hydrogen peroxide was increased by 10.3% compared with that in the control group (Figures 5B, C). It has been reported that the levels of glutathione within brain neurons are reduced in AD patients, which leads to increased sensitivity of the neurons to oxidative damage (Allen et al., 2012). Our RNA-seq analysis indicated that the transcription level of *gsto-1*, a glutathione transferase that is primarily involved in the metabolism of glutathione within cells, was significantly downregulated in *hlh-30* mutants. Paralysis experiments revealed that the paralysis delaying and RO reducing effects of *hlh-30* overexpression were reversed by *gsto-1* RNAi (Figures 5D, E). The augmented antioxidant capacity following *hlh-30* overexpression was also closely associated with *gsto-1* (Figure 5F). These findings indicate that *gsto-1* could potentially contribute to the regulation of oxidative stress and the maintenance of protein homeostasis in worms by *hlh-30*.

### Saikosaponin B2 and hypericin ameliorate the proteostasis imbalance caused by Aβ by promoting HLH-30 translocation to the nucleus and reducing autophagosome accumulation

**FIGURE 6:**
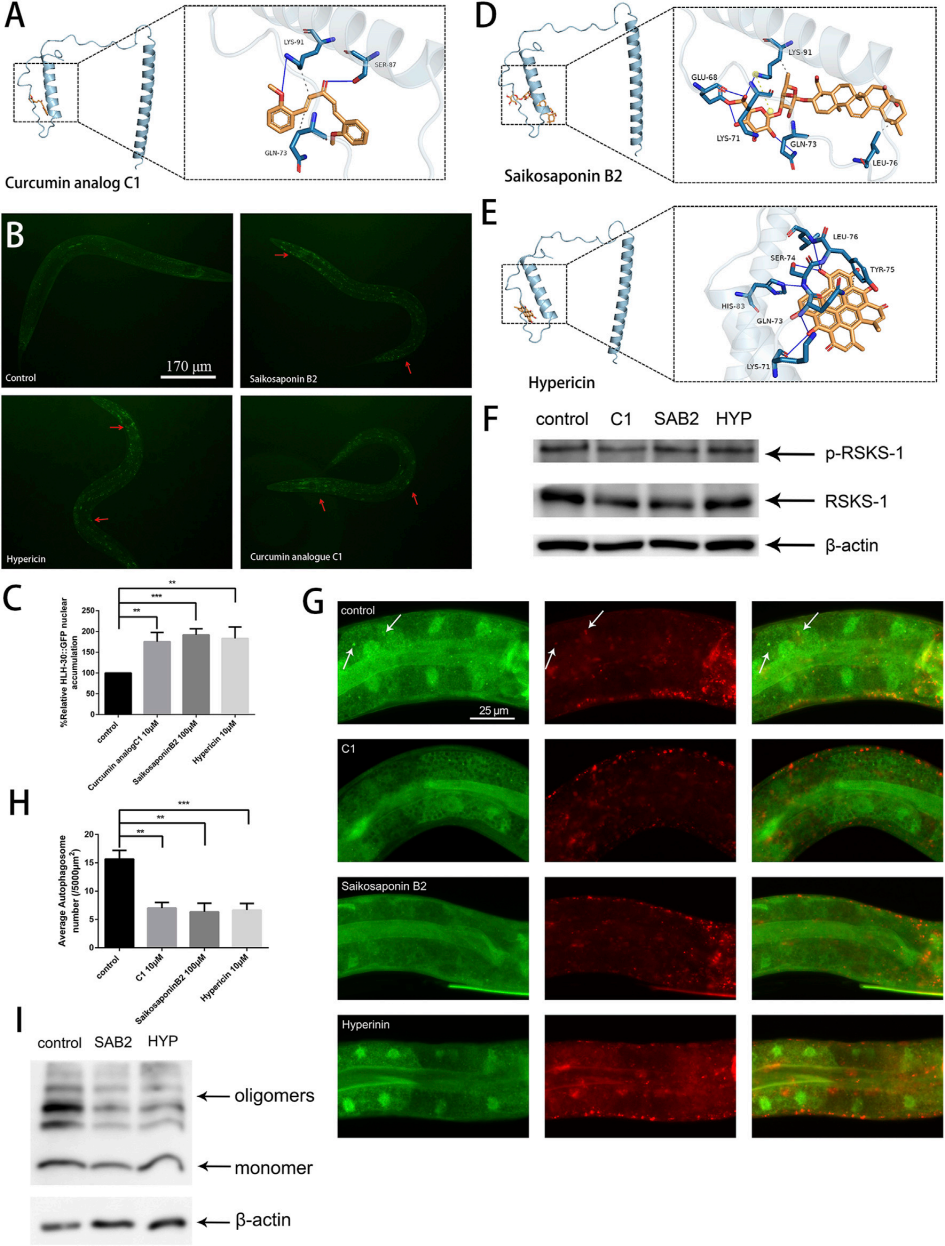
Saikosaponin B2 and hypericin improve protein homeostasis by reducing autophagosome accumulation and alleviating oxidative stress. **(A)** Possible binding sites of the curcumin analog C1. **(B)** GFP fluorescence images of HLH-30 in the nucleus (n = 10 for each group, repeated three times, strain HBW001). **(C)** Fluorescence signal ratio quantification. The HLH-30::GFP accumulation in the control group was defined as 100% for quantitative comparisons. **(D)** Possible sites of action of saikosaponin B2. **(E)** Possible sites of action of hypericin. **(F)** Immunoblot analysis to evaluate the effects of C1 (C1), saikosaponin B2 (SAB2), and hypericin (HYP) on the phosphorylation of RSKS-1 and total RSKS-1. **(G)** Fluorescence microscopy analysis to evaluate the effects of the C1, saikosaponin B2, and hypericin on puncta formation in the PHX3392 strain. **(H)** Scoring of punctae (n = 10 for each group, repeated three times). **(I)** Immunoblot analysis to evaluate the effects of saikosaponin B2 (SAB2) and hypericin (HYP) on Aβ species (strain GMC101). **p < 0.01; ***p < 0.001. Detailed genotypic descriptions of strains HBW001, PHX3636 and GMC101 are listed in Supplementary Material.

TFEB/HLH-30 regulators that do not inhibit the mTOR pathway are preferred as they may have less harmful effects on cells (Song et al., 2016). The curcumin analog C1, a TFEB-specific activator, has been found to significantly reduce Aβ and tau aggregation and improve synaptic and memory functions in different AD animal models (Song et al., 2020). We used C1 as a ligand for molecular docking to identify the active site of TFEB (Figure 6A). Then, based on the predicted active site, we screened thousands of small molecule compounds derived from natural products. Subsequently, we evaluated the impact of the top ten small molecular compounds (Supplementary Table S3), as determined by docking scores, on the paralysis rate of the Aβ strain CL4176 (Supplementary Figure S2).

By combining the results of the HLH-30::GFP nuclear localization and paralysis assays, we identified two active compounds, saikosaponin B2 and hypericin (Figures 6B, C). Molecular docking revealed that the binding sites of saikosaponin B2, hypericin, and the curcumin analog C1 on TFEB were relatively similar (Figures 6A, D, E). By detecting the phosphorylation levels of TOR and its downstream target protein RSKS-1, we confirmed that neither saikosaponin B2 nor hypericin could inhibit TOR activity (Figure 6F). Further, both saikosaponin B2 and hypericin could reduce autophagosome accumulation, maintain the stability of autophagic flux (Figures 6G, H) and promote Aβ degradation in worms (Figure 6I). Our results suggested that targeting TFEB/HLH-30 presents a promising alternative for discovering new treatments for AD.

## Discussion

Previous studies have documented autophagy dysfunction in the brains of Alzheimer’s disease (AD) patients, characterized by the accumulation of autophagosomal vesicles (Yu et al., 2005). However, the underlying causes of this phenomenon remain elusive. Abnormal phosphorylation of the tau protein prevents the fusion of autophagosomes with lysosomes (Hamano et al., 2021). Protein misfolding, immune abnormalities, and loss of lysosomal activity can also lead to impaired autophagy (Cozzi and Ferrari, 2022). In this work, we used transgenic *C. elegans* to explore the association between Aβ and autophagy dysfunction. We found that the expression of Aβ in *C. elegans* activates the mTOR pathway, which inhibits the entry of the transcription factor HLH-30 into the nucleus (Figures 1B–D) and ultimately leads to the accumulation of autophagosomes (Figures 2A–C). These observations provide insight into the mechanisms underlying autophagy dysfunction in the brains of Alzheimer’s disease (AD) patients.

Autophagy is a double-edged sword in neurodegenerative diseases (Zhou and Soukas, 2019). Under normal conditions, autophagy is important for the degradation of misfolded proteins (Chua et al., 2022). However, when autophagy is abnormal, its induction alone increases the metabolic burden on brain cells (Zhang et al., 2021). As a transcription factor that can simultaneously induce autophagy and increase lysosomal activity, TFEB/HLH-30 is a worthy target for study. Here, we discovered that *hlh-30* is the key gene involved in regulating protein homeostasis through our observations of an *hlh-30*-overexpressing strain and an *hlh-30 (syb6883)* mutant strain (Figures 2E, H). Through RNA-seq, we found that *hlh-30* overexpression can significantly increase the level of cathepsin B transcripts and promote lysosomal activity. Cathepsin B has been reported to degrade Aβ (Hook et al., 2020). We identified the critical role of cathepsin B in the regulation of protein homeostasis mediated by HLH-30 through the use of specific cathepsin B inhibitors (Figures 4F, G). The lysosome, a key organelle that degrades misfolded proteins, plays a necessary role in maintaining protein homeostasis. With increasing age, lysosome function gradually weakens, resulting in waste materials and aging organelles in the cell not being effectively removed, which in turn accelerates the aging process of the cell (Colacurcio and Nixon, 2016). In this study, we found that overexpressing *hlh-30* could enhance lysosomal acidification and the expression of cathepsins, which contribute positively to protein homeostasis. It is necessary to consider age-related lysosomal dysfunction when developing treatment strategies for TFEB-related Alzheimer’s disease.

Furthermore, we discovered that the regulation of protein homeostasis by *hlh-30* is dependent on the involvement of *gsto-1* (Figures 5D, F). The gene *gsto-1* encodes glutathione transferase, which is involved in the synthesis and transport of glutathione within cells (Burmeister et al., 2008). Glutathione transferase is also an important factor in the crosstalk between apoptosis and autophagy (Mazari et al., 2023). However, there is insufficient research on the relationship between *hlh-30* and *gsto-1*. Moreover, the interactions among apoptosis, autophagy dysfunction and severe oxidative stress contribute greatly to the difficulty of studying the disease mechanisms of AD (Twarowski and Herbet, 2023); therefore, finding suitable models and research methods is the key to advancing the study of these factors.

The known activators of TFEB/HLH-30 are mainly inhibitors of mTOR, such as rapamycin. As a major regulator of cell growth and metabolism, mTOR is involved in a wide range of biological functions (Saxton and Sabatini, 2017). Therefore, identifying TFEB-specific activators is a promising strategy, as it may offer reduced cytotoxicity to cells (Song et al., 2021). We found that two natural products, saikosaponin B2 and hypericin, have potential as TFEB activators. The binding sites of saikosaponin B2, hypericin, and the curcumin analog C1 are located at the N-terminus of TFEB (Figures 6A, D, E), suggesting that the development of activators targeting TFEB should focus on the N-terminal protein structure of TFEB. Furthermore, both saikosaponin B2 and hypericin improved autophagy dysfunction and promoted the degradation of Aβ (Figures 6G, I) without inhibiting TOR activity (Figure 6F). In summary, our findings support a close association between inhibited nuclear entry of HLH-30/TFEB and autophagy dysfunction in AD and indicate that identifying TFEB-specific activators is a promising strategy for treating AD.

## Conclusion

We report that Aβ-induced autophagy dysfunction in *C. elegans* may be associated with the inhibition of nuclear translocation of the transcription factor HLH-30. This could lead to a reduction in the expression of the membrane fusion gene *syx-17*, which is a key molecular event in Aβ-induced autophagy dysfunction. Overexpression of the *hlh-30* gene appears to enhance autophagy activity and may decrease autophagosome accumulation, potentially promoting Aβ degradation. In addition, two potential HLH-30/TFEB activators, saikosaponin B2 and hypericin, were found by molecular docking and demonstrated to promote the nuclear entry of HLH-30, thereby enhancing autophagy and facilitating the degradation of Aβ.

## Data Availability

The raw data supporting the findings of the study are publicly available and can be accessed through the NCBI Gene Expression Omnibus (GEO) database with the accession number GSE272146: https://www.ncbi.nlm.nih.gov/geo/query/acc.cgi?acc=GSE272146.

## Abbreviations

AD, Alzheimer’s disease; ALP, Autophagy lysosome pathway; Aβ, Amyloid beta; C1, Curcumin analog C1; *C. elegans*, *Caenorhabditis elegans*; GFP, Green fluorescent protein; mCherry, Cherry fluorescent protein; mTOR, Mammalian target of rapamycin; RNAi, RNA interference; ROS, Reactive oxygen species; S6K, Substrate S6 kinase; TFEB, Transcription factor EB; TOR, Target of rapamycin; v-ATPase, Vacuolar ATPase.
