# Supplementary material for: HLH-30/TFEB modulates autophagy to improve proteostasis in Aβ transgenic Caenorhabditis elegans
Source: Front Pharmacol. 2024 Aug 30;15:1433030. doi: 10.3389/fphar.2024.1433030 (PMC11392864; doi:10.3389/fphar.2024.1433030)
Supplement: Supplementary file 1 [file DataSheet1.docx]

HLH-30/TFEB Modulates Autophagy to Improve Proteostasis in Aβ Transgenic *Caenorhabditis elegans*

Hongru Lin^[1]^, Chen Zhang^[1]^, Yehui Gao^[1]^, Yi Zhou^[3]^, Botian Ma^[1]^, Jinyun Jiang^[1]^, Xue Long^[1]^, Nuerziya Yimamu^[1]^,Kaixin Zhong^[1]^, Yingzi Li^[1]^, Xianghuan Cui^[1]^*, Hongbing Wang^[1,2]^*

[1] Institute for Regenerative Medicine, Shanghai East Hospital, School of Life Sciences and Technology, Tongji University, Shanghai 200092, China

[2] Tongji Alpha Natural Medicine Research Institute, Tongji University, Shanghai 200070, China

[3] Department of Anesthesiology, The First Affiliated Hospital of Naval Medical University, Shanghai 200433, China

*Corresponding author:

*E-mail address:* [*hbwang@tongji.edu.cn*](mailto:hbwang@tongji.edu.cn) *(H.B.Wang);* [*cuixh@tongji.edu.cn*](mailto:cuixh@tongji.edu.cn) *(X.H. Cui)*

Supplementary material


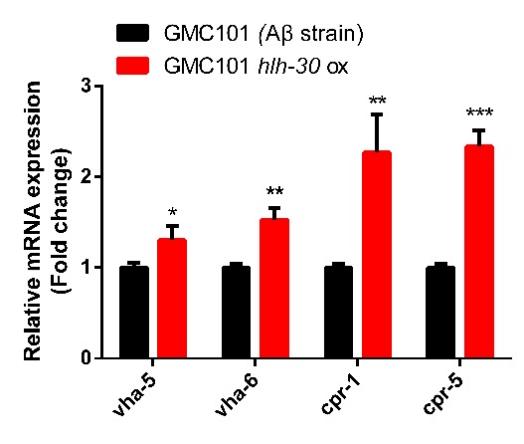


Figure S1. Effects of *hlh-30* overexpression on the transcript levels of the v-ATPase and cathepsin B genes. **p* < 0.05; ***p* < 0.01; ****p* < 0.001


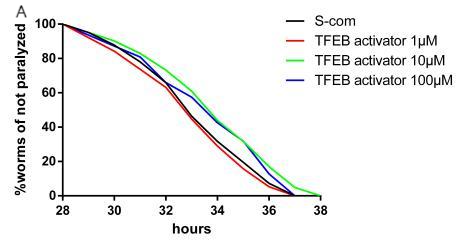

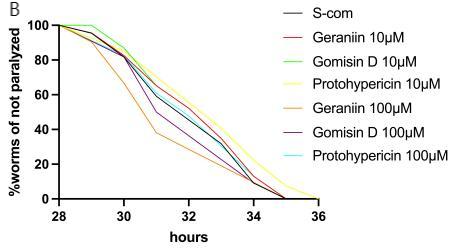


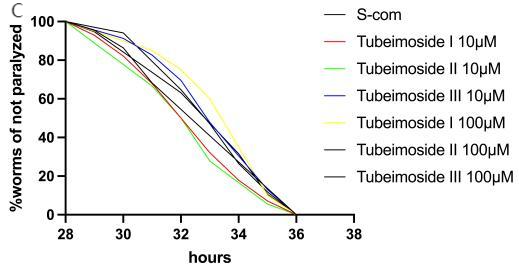

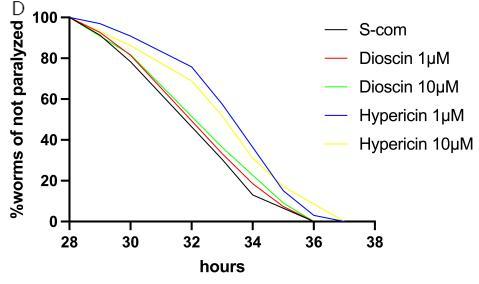


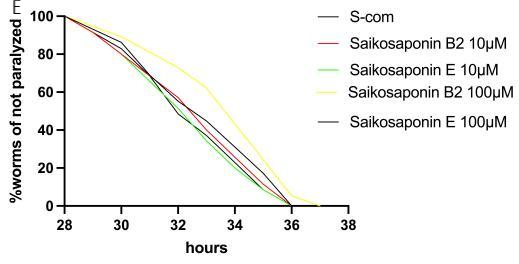


Figure S2. Effect of a TFEB activator (curcumin analog C1) and small molecule compounds identified by molecular docking screen on the paralysis rate of CL4176.

Table S1 Strain name List:

| Strain | Relevant genotype | Source |
| --- | --- | --- |
| CL4176 | [(pAF29) myo-3p::Aβ1–42 + (pRF4) rol-6 (su1006)] | CGC |
| GMC101 | [unc-54p::A-beta-1-42::unc-54 3′-UTR + mtl-2p::GFP] | CGC |
| CL2122 | [(pPD30.38) unc-54 (vector) + (pCL26) mtl-2:GFP] | CGC |
| JIN1821 | [hlh-30p::hlh-30::GFP] | Professor Ben Zhou |
| PHX3392 | [unc-54p::Aβ1–42::unc-54 3’-UTR + mtl-2p::GFP]; Ex[Plgg-1::mCherry::GFP::lgg-1 3’-UTR; pRF4] | Suny biotech |
| PHX3636 | [(pPD30.38) unc-54 (vector) + (pCL26) mtl-2:GFP] ; Ex[Plgg-1::mCherry::GFP::lgg-1 3’-UTR; pRF4] | Suny biotech |
| PHX6883 | [hlh-30(syb6883) dvIs100] | Suny biotech |
| HBW001 | [(pAF29) myo-3p::Aβ1–42 + (pRF4) rol-6 (su1006)]; [hlh-30p::hlh-30::GFP] | This study |
| HBW002 | [unc-54p::Aβ1–42::unc-54 3’-UTR + mtl-2p::GFP]; [hlh-30p::hlh-30::GFP] | This study |
| HBW003 | [unc-54p::Aβ1–42::unc-54 3’-UTR + mtl-2p::GFP]; Ex[Plgg-1::mCherry::GFP::lgg-1 3’UTR; pRF4]; [hlh-30p::hlh-30::GFP] | This study |
| HBW004 | [hlh-30(syb6883)dvIs100]; Ex[Plgg-1::mCherry::GFP::lgg-1 3’UTR; pRF4] | This study |

Table S2 Primer List:

| lmtr-2 F | GGAAGGATTGCTCCTTGCCT |
| --- | --- |
| lmtr-2 R | ACTCTTGTCGGCCTTGACTG |
| lmtr-3 F | GCATTGTACGAGTCCCACCA |
| lmtr-3 R | TGAGAGCAGCCACCACATAA |
| lmtr-5 F | CGGAAGGTGTCAGGCTTCAT |
| lmtr-5 R | ATTCTTCGCATCGGCGATCA |
| raga-1 F | CGACTCGTTCACCCAGAACA |
| raga-1 R | ATAACTGGAATGCGGGGCAA |
| ragc-1 F | TTACCGCTACGGAATCGACG |
| ragc-1 R | TTCCGCTTCTCTTGTGTCCC |
| vha-5 F | CTTCATGGAAACGCGACTGT |
| vha-5 R | CGGTAACGAACACCATGTGC |
| vha-6 F | CTTGCTCACGCACAGCTTTC |
| vha-6 F | TGAAGGAAGGCGGAAAGACC |
| cpr-1 F | TTATCCGTGACCAAGCCACC |
| cpr-1 R | TGCATGGGGCAATTGGGTAT |
| cpr-5 F | CTCCGACGCTATTCCAGACC |
| cpr-5 R | GCGTAGGCGGTAGATCCAAA |
| actin-1 F | CGGAGGAACCACCATGTACC |
| actin-1 R | TAGAAAGCTGGTGGTGACGAT |
| lmp-2 F | CCAGTGACAGTGAGAAGGACG |
| lmp-2 R | TGCATGATGACACCCCAGTG |
| rab-7 F | TTCCTCACACGCGACGTAAA |
| rab-7 R | CGGCTCCACGATAAAAAGCG |
| syx-17 F | TGTTGGATTGGCAGCTGGAT |
| syx-17 R | CCCGGTGTAGATTGTAGCGA |

Table S3 Autodock affinity score

| Natural product | affinity(kcal/mol) |
| --- | --- |
| Geraniin | -15.2 |
| Tubeimoside III | -14.5 |
| Tubeimoside II | -14.4 |
| Tubeimoside I | -14.2 |
| Gomisin D | -13.3 |
| Hypericin | -12.9 |
| Saikosaponin E | -12.9 |
| Protohypericin | -12.9 |
| Saikosaponin B2 | -12.6 |
| Dioscin | -12.6 |
